# Combination antiangiogenesis therapy with marimastat, captopril and fragmin in patients with advanced cancer

**DOI:** 10.1038/sj.bjc.6601897

**Published:** 2004-05-25

**Authors:** P H Jones, K Christodoulos, N Dobbs, P Thavasu, F Balkwill, A D Blann, G J Caine, S Kumar, A J Kakkar, N Gompertz, D C Talbot, T S Ganesan, A L Harris

**Affiliations:** 1 Cancer Research UK Medical Oncology Unit, Churchill Hospital, Headington, Oxford OX3 7LJ, UK; 2 Cancer Research UK, 44 Lincoln's Inn Fields, London, UK; 3 Cancer Research UK, Translational Oncology Laboratory, Barts and The London, Queen Mary's School of Medicine & Dentistry, 3rd Floor John Vane Science Centre, Charterhouse Square, London, UK; 4 Thrombosis and Vascular Biology Unit, University Department of Medicine, City Hospital, Birmingham, UK; 5 Tumour Biology Laboratory, Christie Hospital, Wilmslow Road, Withington, Manchester, UK; 6 Department of Surgery, Imperial College, London UK

**Keywords:** metalloprotease inhibitor, heparin, tumour necrosis factor

## Abstract

Marimastat, low molecular weight heparins and captopril have antiangiogenic activity *in vitro* and in animal models. We studied the safety and efficacy of the combination of these drugs in patients with advanced cancer. In all, 50 patients were enrolled. Captopril was given orally at a dose of 50 mg bd daily. Fragmin was administered as a daily subcutaneous injection of 200 units kg^−1^ for the first 28 days and 5000 units thereafter. Marimastat was given at 10 mg bd orally. Serum, plasma and urinary angiogenic factors were measured at baseline and after 1 month of treatment. Inhibition of release of tumour necrosis factor alpha (TNF-alpha) from peripheral lymphocytes was used as a surrogate pharmacodynamic end point. There was one case of haemorrhagic stroke and one upper gastrointestinal haemorrhage. The commonest toxicity was myalgia. One of 10 patients with renal cancer had a partial response, and three patients had a prolonged period of stable disease. The treatment significantly inhibited phytohaemagglutinin (PHA)-stimulated TNF-alpha release from patient's lymphocytes. The combination of marimastat, fragmin and captopril is well tolerated and has *in vivo* activity. Inhibition of PHA-stimulated TNF-alpha release from lymphocytes is a surrogate pharmacodynamic marker of metalloprotease inhibition.

Tumour angiogenesis involves multiple signalling pathways that provide potential therapeutic targets to inhibit tumour growth and metastasis (Fox *et al*, 2001). Most trials of antiangiogenic agents to date have used single agents. In an attempt to improve the efficacy of angiogenic blockade, we describe a phase 2 clinical trial of a combination of captopril, marimastat and low molecular weight heparin, each of which has antiangiogenic activity *in vitro* and in *in vivo* models. The aim was to assess the response to the combination, including a predefined duration of stable disease (SD), as angiogenic agents may have not necessarily caused objective regression but still have effects on tumour growth. We also planned to relate relevant mechanistic pathways before and during therapy to assess whether early changes were related to later anticancer effects. We also investigated whether inhibition of release of tumour necrosis factor alpha (TNF-alpha) from phytohaemagglutinin (PHA)-stimulated lymphocytes is a useful surrogate pharmacodynamic marker for matrix metalloprotease inhibition in patients treated with this drug combination.

Captopril, an angiotensin-converting enzyme (ACE) inhibitor, has both antiangiogenic and antitumour activity in rodent models (Volpert *et al*, 1996) and inhibits the growth of human renal carcinoma xenografts (Hii *et al*, 1998). Epidemiological studies suggest that the incidence of cancer and cancer deaths are lower in hypertensive patients taking ACE inhibitors than in those treated with other classes of antihypertensives (Lever *et al*, 1998). Captopril has been shown to inhibit matrix metalloproteases 2 and 9, which are involved in the angiogenic process (Prontera *et al*, 1999).

The rationale for the combination of captopril and the matrix metalloprotease inhibitor (MMPI) marimastat in this study comes from pre-clinical studies using the murine Lewis lung carcinoma model (Prontera *et al*, 1999). When captopril was administered with the MMPI Batimastat, the combination had synergistic activity both in inhibition of matrix metalloproteases 2 and 9 in the tumour cells and in blocking tumour growth and metastasis. Batimastat is not suitable for oral administration, so the board spectrum orally available MMPI, marimastat, is used in combination with captopril (Wojtowicz-Praga *et al*, 1998; Macaulay *et al*, 1999).

As captopril and marimastat are synergistic inhibitors of common target enzymes, we wanted to add a third antiangiogenic agent that is not thought to work via MMP inhibition, in an attempt to target multiple stages of the angiogenic pathway. Low molecular weight heparins (LWMH) are antiangiogenic in model systems, and prolong survival in animal models (Folkman *et al*, 1983). Their mode of action is unclear but may include inhibition of proangiogenic factors such as fibroblast growth factor 2 and angiogenin (Folkman, 1983; Norrby and Ostergaard, 1996; Soncin *et al*, 1997). Low molecular weight heparins have been shown to improve the survival of cancer patients in retrospective analysis of randomised trials investigating the role of LWMH in treatment of deep vein thrombosis (Green *et al*, 1992; Kakkar and Williamson, 1999; von Tempelhoff *et al*, 2000).

Early-phase studies of antiangiogenic agents have been hampered by the lack of pharmacodynamic end points to monitor the effectiveness of therapy (Gelmon *et al*, 1999). A surrogate pharmacodynamic marker for MMPI therapy is TNF-alpha converting enzyme (TACE), which is inhibited by metalloprotease inhibitors in preclinical studies (Gearing *et al*, 1994; McGeehan *et al*, 1994). Both captopril and marimastat have been shown to inhibit TNF-alpha release from stimulated lymphocytes (Peeters *et al*, 1998). Marimastat acts so as to inhibit TNF-alpha release from stimulated lymphocytes in a mouse model (Tsuji *et al*, 2002). We assayed TNF-alpha release after PHA treatment in blood samples (Thavasu *et al*, 1999; Propper *et al*, 2001), before, during and after treatment to see if we could observe inhibition during MMPI treatment. In addition, the levels of circulating and urinary angiogenic factors were measured to determine if these varied with therapy and could be related to response.

## PATIENTS AND METHODS

Patients aged over 18 years with WHO performance status 0–2 and histologically proven advanced malignancy refractory to conventional treatment who gave written informed consent were eligible for the trial. The trial was approved by the Oxford Research Ethics Committee according to principles in the Helsinki Declaration. Patients were required to have adequate haematological, renal and hepatic function and have evaluable or measurable disease. The exclusion criteria for the trial were a second malignancy, pregnancy or lactation, pregnancy, inadequate contraception, treatment with anti-cancer agents in the previous 4 weeks (6 weeks in the case of nitrosureas and mitomycin), serious systemic illness and patients with contraindications to the study drugs. Thus, patients with previous bleeding disorder, including heparin, induced thrombocytopaenia, a history of gastrointestinal haemorrhage or uncontrolled peptic ulceration, renal artery stenosis, cardiac outflow obstruction, severe arteriosclerosis, or recent treatment with procainamide or allopurinol. Patients were not allowed to receive aspirin, warfarin, lithium, allopurinol or procainamide while on study.

The trial was carried out in two stages, expecting a lower response for antiangiogenic therapy than conventional chemotherapy. To detect the activity level of 10% including a SD of 4 months or longer, Simon two-stage design (alpha=0.05 and beta=0.20%) required enrolment of 18 patients to the first stage. If there were at least three responders in the 18 evaluable patients, then an additional 25 evaluable patients would be enrolled (total of 43 patients). To allow for patient dropouts or other reasons that would declare a patient not evaluable, it was recommended that a total of 50 patients be enrolled. As we developed the PHA assay, and there was sufficient response in the first cohort, a subsequent group of patients was recruited to prospectively analyse the effects of the therapy on an *in vivo* pharmacodynamic end point and relation to SD.

Drugs were administered according to the following schedule. A test dose of 12.5 mg of captopril was given orally. If patients tolerated the first dose, then they continued on captopril taking 12.5 mg twice daily for 1 week, followed by 25 mg twice daily for 1 week, after which they took 50 mg twice daily for the duration of the study. Marimastat was administered orally at a dose of 10 mg twice daily for the duration of the study. This dose was chosen based on pharmacokinetic data from a phase 1 trial of marimastat in patients with advanced cancer, which suggested that this dose would generate plasma concentrations in the range required to inhibit the target enzymes, MMP2 and MMP9, while minimising the incidence of musculoskeletal side effects (Wojtowicz-Praga *et al*, 1998). This dose level has been used in several phase II and III trials of marimastat as a single agent (Bramhall *et al*, 2002; Miller *et al*, 2002; Shepherd *et al*, 2002). Fragmin was administered by a once daily subcutaneous injection at a dose of 200 units kg^−1^ for 4 weeks, after which the dose was reduced to 5000 units per day for the duration of the study. The dose of fragmin was reduced to minimise both the risk of haemorrhage and severe bruising at injection sites. In addition, prolonged administration of heparins at high doses may result in osteoporosis in animal models (Shaughnessy *et al*, 1995). Toxicities were graded according to the NCI common toxicity criteria. Patients with grade 3 or 4 toxicity were withdrawn from the study. Full details of dose reductions for grade 1 or 2 toxicities are available from the authors. Briefly, for grade 1 hypotension the dose of captopril was halved, for grade 2 hypotension captopril was omitted, but Fragmin and marimastat continued. For myalgia grade 2, trial medication was discontinued for 1 week and then restarted with half-dose marimastat if symptoms had resolved or with marimastat omitted if symptoms persisted.

Evaluation of the response to treatment was by appropriate imaging after 16 weeks. Patients with SD or response were permitted to remain on study until they developed unacceptable toxicity or symptomatic disease progression. Complete response (CR) was defined as the disappearance of all known disease, determined by two observations not less than 4 weeks apart. Partial response (PR) was a decrease of at least 50% of the sum of the products of the largest perpendicular diameters of all bidimensionally measurable lesions, as determined by two observations not less than 4 weeks apart. It was not necessary for all lesions to have regressed to qualify for PR, but no lesion could have progressed and no new lesion appeared. SD was a <50% decrease and <25% increase in the sum of the products of the largest perpendicular diameters of all bidimensionally measurable lesions, in the absence of any new lesions, lasting for 4 months or longer from start of therapy. Progressive disease (PD) is a ⩾25% increase in the size of at least one bidimensionally measurable lesion or appearance of a new lesion. Tumour markers were not used to assess response.

### Assays of angiogenic factors

Samples of blood and urine were analysed for expression of angiogenic factors at baseline and at 28 days using enzyme-linked immunosorbent assays. Plasma levels of active plasminogen activator inhibitor 1 (PAI-1) were assayed with an actibind-PAI-1 kit (Tachnoclone, Dorking, UK). ELISA assays were obtained from the following suppliers and used according to the manufacturer's instructions: Human Endostatin Protein Accucyte Kit, CN Biosciences, Nottingham, UK, soluble E selectin, basic fibroblast growth factor, soluble Vascular cell adhesion molecule-1 and vascular endothelial growth factor, R and D Systems, Abingdon, UK, and von Willibrand factor, Alpha Labs, Hants, UK. In total, 22 age- and sex-matched healthy controls, who were taking no prescription medications and were free of neoplastic disease, diabetes, cardiovascular or connective tissue disease, supplied samples for controls. CD105, TGF*β*1 and CD105/TGFb1 complexes were determined by immunoassay as described, with control samples taken from 40 healthy volunteers (Li *et al*, 2000).

Baseline samples were compared to on therapy results by two-tailed paired *t*-tests, with a *P*-value <0.05 taken as significant.

### Measurement of PHA-stimulated TNF-alpha release from patient lymphocytes (whole blood cytokine assay)

Whole blood samples were collected into tubes containing preservative-free heparin (30 units ml^−1^ of blood) and PHA (2 mg ml^−1^ of blood, Murex Biotech Ltd, Dartford, UK) immediately pre-treatment and at 24 h and 7 days after commencing treatment. The assays were carried out as we previously described (Thavasu *et al*, 1992).

**Figure 1:**
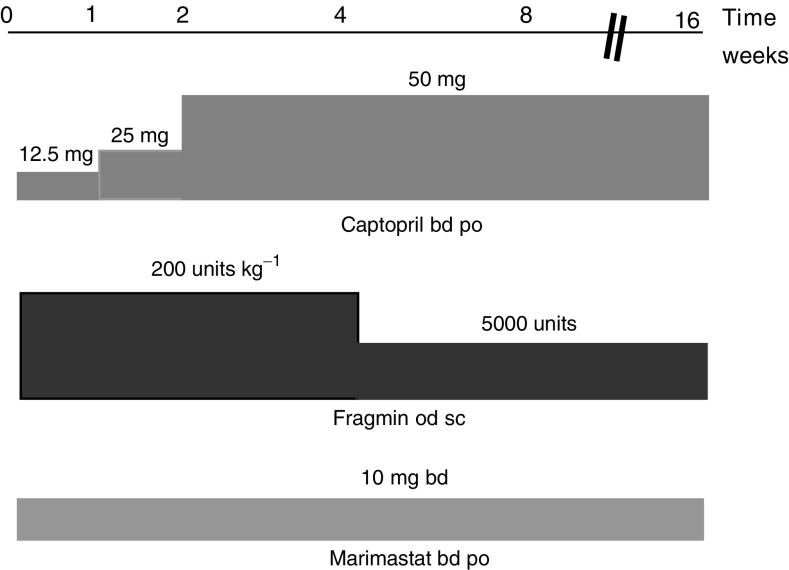
Dosage regimen for COMBAT study.

The dose of captopril was increased after the TNF-alpha release sample was taken. This is clarified in Figure 1

, which summarises the dose schedule. The 1-week sample reflects the level of dose inhibition achieved by a captopril dose of 12.5 mg twice daily in combination with marimastat 10 mg daily and fragmin 200 units kg^−1^.

Control samples were collected at the same time points into tubes containing heparin alone. All samples were collected and processed under sterile, pyrogen-free conditions and were incubated at 37°C in a 5% CO_2_ saturated humidified incubator. After 24 h of *ex vivo* stimulation with PHA, the blood samples were cool spun and the plasma flash frozen at −20°C.

Tumour necrosis factor alpha levels were measured using IRMA kits (Biosource Europe, Brussels, Belgium). The TNF-alpha IRMA range was from 15–500 pg ml^−1^. The assay was calibrated with the international reference preparation (87/650, National Institute of Biological Standards and Controls, Potters Bar, Herts, UK) and was used at detection limits of 20 pg ml^−1^ plasma. The calibration, standardisation and the assay format were specifically developed for measuring TNF-alpha in plasma samples (Thavasu *et al*, 1999; Propper *et al*, 2001).

## RESULTS

### Patients

A total of 50 patients were enrolled in the study. The characteristics of the patients are shown in Table 1

**Table 1:** Patient characteristics

Number of patients	50
Male	36
Female	14
Mean age (range), years	59.1 (27–80)
	
*Diagnosis*	
Renal carcinoma	14
Prostate carcinoma	4
Colorectal carcinoma	10
Gastric carcinoma	4
Sarcoma	4
Breast carcinoma	2
Melanoma	2
Mesothelioma	2
Cervical carcinoma	2
Adrenal carcinoma	1
Parotid carcinoma	1
Small cell lung carcinoma	1
Bladder carcinoma	1
Carcinoma – unknown primary	2
	
*WHO performance status*	
0	22
1	21
2	7

.

### Toxicity

Two patients died while on study. A 28-year-old woman with locally advanced cervical carcinoma refractory to radiotherapy and chemotherapy developed profuse rectal bleeding, requiring a 6-unit blood transfusion after 18 days on study. Study medication was discontinued immediately. She had a normal prothrombin time and activated partial thromboplastin time, but the haemorrhage continued and she died 6 days later. A second patient with advanced transitional cell carcinoma of the renal pelvis and a previous history of pulmonary emboli became non-specifically unwell at home after 36 days on study. Trial medication was stopped, but the next day she was admitted to a local hospital with circulatory collapse and died shortly afterwards. A post mortem was not carried out.

The principle toxicity of the treatment was major bleeding events. In addition to the patient who died from uncontrolled haemorrhage, one patient suffered a nonfatal haemorrhagic stroke and another had an upper gastrointestinal haemorrhage, requiring a 3-unit blood transfusion. Muscle and joint pains were common, with grade 2 or 3 toxicity developing after mean of 69 days on study in 46% patients (Table 2

**Table 2:** Toxicity of captopril, marimastat and fragmin

	**Grade**				
**Toxicity**	**0**	**1**	**2**	**3**	**4**
Rectal bleeding	0	0	0	0	1
Haematemesis	0	0	0	0	1
CNS haemorrhage	0	0	0	0	1
Myalgia	8	20	16	7	0
Elevation of aspartate transaminase	26	18	5	1	0
Hypotension	35	14	2	0	0
Taste change	26	21	3	0	0
Cough	32	13	1	2	0

One patient died from haemorrhage and one patient died with circulatory collapse of unknown cause.

). Musculoskeletal side effects responded to dose reduction or withdrawal of marimastat. In all, 21 patients (42%) required dose reduction of Marimastat. Mild elevation of aspartate transaminase, taste changes and cough were seen. Grade 2 hypotension occurred in two patients, but there was no fall in blood pressure in any other patients.

### Response to treatment

**Figure 2:**
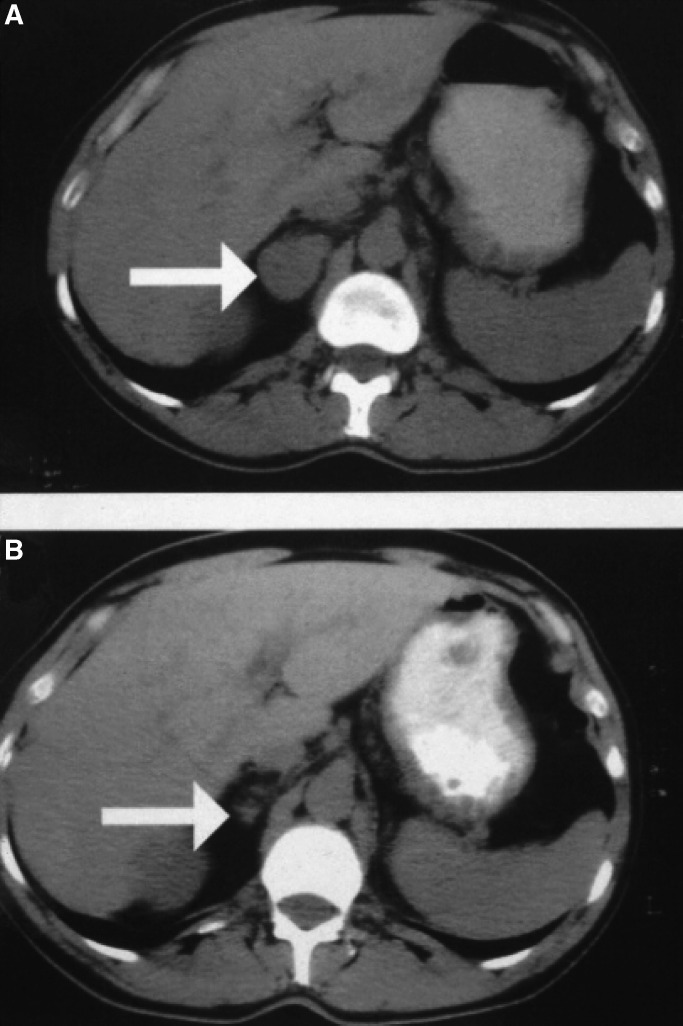
Response to treatment in a patient with renal carcinoma. CT sections imaging the right adrenal gland (arrowed) in a patient with renal carcinoma. (**A**) Baseline scan, (**B**) scan after 16 weeks of treatment.

Response to treatment was evaluable in 45 patients. Of the five nonevaluable patients, one withdrew from the study from personal preference, two withdrew because of toxicity, and two died while on trial treatment. One of the 10 patients with renal carcinoma had a PR at 16 weeks, which was maintained for 503 days (Figure 2

). There were no other responses. Three patients with renal carcinoma had SD at 16 weeks; the durations of disease stabilisation for these patients were 204, 219 and 337 days. One patient with colorectal carcinoma had radiological SD at 16 weeks on a CT scan of the liver, but progressed clinically 7 days after the scan. The remaining 40 evaluable patients had PD at or before 16 weeks.

### Tumour necrosis factor alpha release from PHA-stimulated lymphocytes

**Figure 3:**
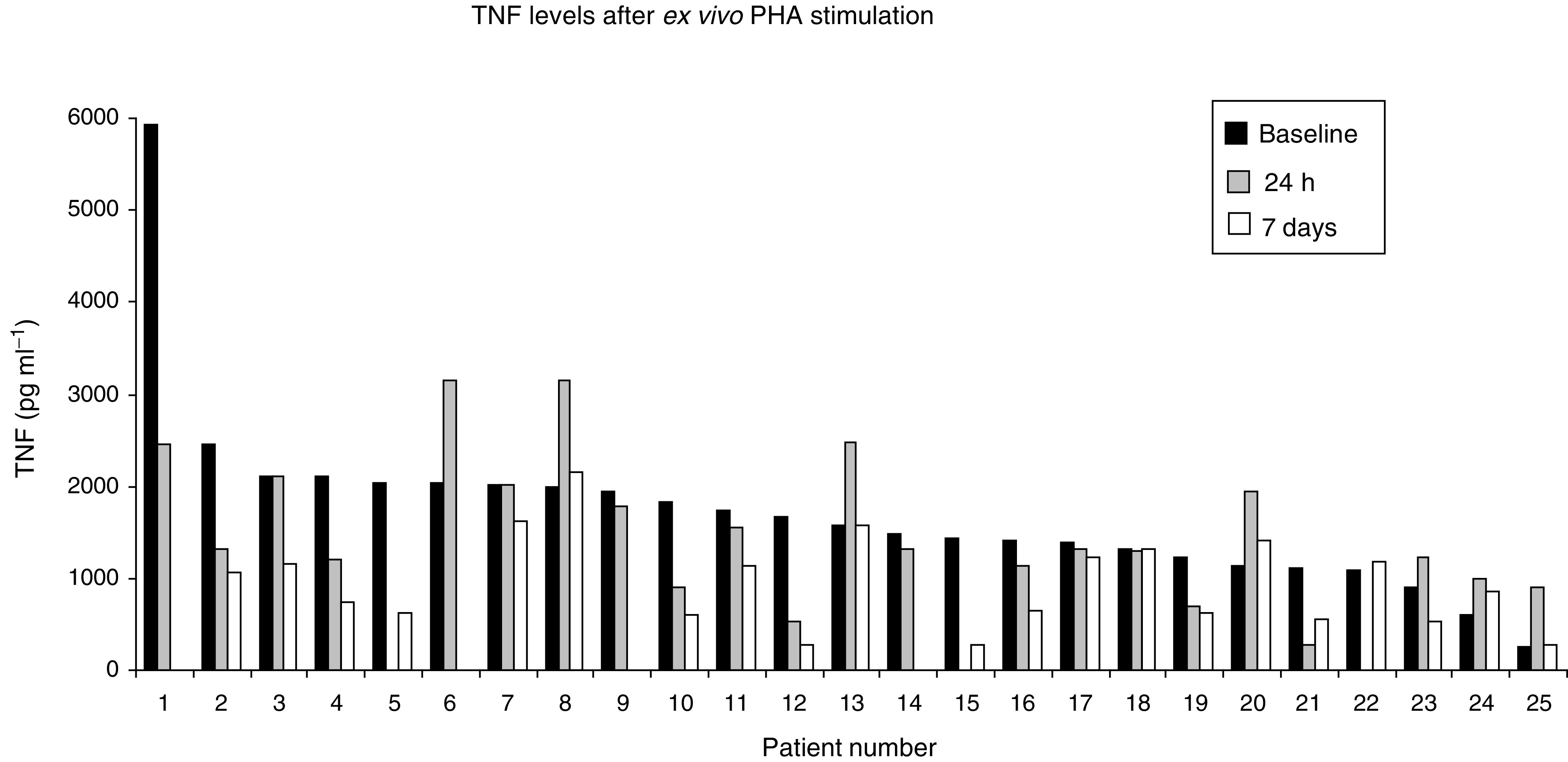
Tumour necrosis factor alpha release from patients' lymphocytes stimulated *ex vivo* with PHA. Blood samples were taken from patients at baseline, after 24 h and 1 week of treatment, and incubated with phytohaemagglutinin for 24 h prior to analysis for TNF-alpha release.

Tumour necrosis factor alpha release from PHA-stimulated lymphocytes was measured *ex vivo* in samples of whole blood. Blood samples, drawn at baseline and after 24 h and 7 days on study, were incubated at 37°C in the presence or absence of PHA as described in Materials and methods. There was no change in lymphocyte counts between baseline and 7 days (mean (standard deviation) 1.1 (0.5) at baseline and 1.2(0.5) at 7 days). Tumour necrosis factor alpha production following 24 h incubation with PHA was assayed at baseline and at 24 h and 7 days in 22 and 21 patients, respectively. Levels of TNF-alpha released were significantly inhibited in the samples taken after 7 days of treatment, as shown in Figure 3

. The mean value of TNF-alpha post stimulation at baseline was 1707±1041 (s.d.) pg ml^−1^, while at 7 days this had fallen to 907±501 pg ml^−1^, *P*<0.001 by two-tailed paired *t*-test. Tumour necrosis factor alpha release from control samples incubated in the absence of PHA was low in comparison to samples treated with PHA and showed no change in TNF-alpha levels with treatment (mean baseline control TNF-alpha release 26 pg ml^−1^, mean day 7 control TNF-alpha release=25 pg ml^−1^). There was no relationship between the baseline levels of TNF-alpha release and the degree of inhibition produced by Marimastat.

### Angiogenic markers

The descriptive statistics of assays of serum, plasma and urine for proteins involved in angiogenesis are presented in Table 3

**Table 3:** Changes in levels of angiogenic factors during trial treatment

**Angiogenic factor**	**Control median (range)**	***n***	**0 days median**	**28 days median**	**56 days median**	P* (0 *vs* 28 days)	P* (0 *vs* 56 days)
Von Willibrand Factor (iu dl^−1^)	91 (34–153)	47	140 (94–212)	150 (77–215)	147 (108–222)	NS	0.004
S oluble V-CAM (ng ml^−1^)	470 (170–860)	47	560 (320–1030)	610 (320–1210)	623 (320–1200)	0.009	0.01
Soluble E Selectin (ng ml^−1^)	38.5 (11–100)	47	58 (18–280)	50 (17–350)	49 (15–420)	0.0052	NS
Serum VEGF (pg ml^−1^)	77.5 (18–8000)	42	150 (10–195,000)	150 (15–270,000)	ND	NS	ND
Plasma VEGF (pg ml^−1^)	ND	42	95 (9–1,800,000)	110 (10–1,900,000)	ND	NS	ND
Endostatin (ng ml^−1^)	7.75 (1.5–24)	47	14 (3.8–49)	15 (3.4–48)	13 (3.2 –45)	NS	NS
bFGF (pg ml^−1^)	2 (1.9)	47	2 (0.2–8)	2.1 (0.2–11.2)	2.25 (0.2–13)	NS	NS
Plasminogen activator inhibitor1 (units ml^−1^)	7 (1.8–22.6))	47	11 (3.2–38.5)	10.8 (2.5–40)	12.8 (3.6–80)	NS	NS
CD105 (ng ml^−1^)	0.88 (0.07–25)	50	1.02 (0–15.7)	0.99 (0–3.8)	0.94 (0–25)	0.01	0.03
TGF *β*1 (pg ml^−1^)	41 (0–202)	50	25 (0–384)	27 (0–327)	28.5 (0–202)	NS	NS
CD105/TGF *β*1 complex (units ml^−1^)	0 (0–2.7)	50	0.6 (0–7)	0.5 (0–2.3)	0.4 (0–5.8)	NS	0.007
Anti Xa (IU ml^−1^)	ND	50	0.02 (0–0.1)	0.2 (0–0.83)	0.07 (0–0.5)	<0.0001	<0.0001
Thrombin-antithrombin complex (*μ*g l^−1^)	ND	49	1.69 (0.01–61)	2.27 (0.01–55)	1.61 (0.01–48)	NS	NS
Activated Factor VII (mU ml^−1^)	ND	50	73.3 (41–144)	66.2 (22–149)	66.8 (32–178)	NS	NS
Thrombomodulin (ng ml^−1^)	ND	50	3.25 (0.8–47)	3.19 (1–14)	3.01 (1–76)	NS	NS

ND=not done;

* =values calculated by sign rank test; *n*=number of patients assayed;

NS=not significant (*P*>0.05).

. The baseline values of soluble E selectin, endostatin, soluble VCAM, Von Willibrand factor and plasminogen activator-1 were significantly different from age- and sex-matched healthy controls (all *P*<0.01). Intrapatient variability was small, but paired significance tests revealed that there was a statistically significant rise in the level of Von Willibrand factor and V-CAM and statistically significant falls in the values of CD105, CD105/TGF*β*1 complex and E Selectin. Though statistically significant, the magnitude of these changes was small (see Table 3). Anti Xa assays were significantly elevated at 1 and 2 months (3–10-fold), consistent with the patients receiving Fragmin therapy.

We also analysed whether changes in the level of angiogenic factors on treatment correlated with outcome (PR or SD *vs* PD). The levels of TGF*β*1were significantly different after 2 months, comparing the SD+PR and PD groups (median (range) SD+PR 41(0–202), PD 21 (0–166), *P*=0.046). Endostatin levels were higher in patients with PD at 2 months (median (range) SD+PR 11.7(4.9–27), PD 15.25 (3.2–45), *P*=0.010). Finally, we examined whether the baseline levels of angiogenic factors were predictive of outcome. The baseline level of endostatin was higher in patients who went on to have PD than in those who had SD or PR at evaluation (median (range) PD=15.3 (3.2–45), SD+PR=11.7 (4.9–27) *P*=0.027). In contrast, baseline levels of activated factor VII were lower in patients who went on to progess (median (range) PD=68 (41–114), SD+PR=80 (53–144), *P*=0.01). Levels of activated factor VII were not influenced by treatment. There were no other significant differences for the markers assayed between patients with PD or SD+PR.

## DISCUSSION

This study is the first reported trial to evaluate a combination of agents with antiangiogenic activity in model systems as opposed to single agents. The synergy between matrix metalloprotease inhibitors and captopril *in vitro* supports their use in combination in this study. As Fragmin is thought to exert its antiangiogenic effects by a different mechanism and is well tolerated in cancer patients, we hoped that its inclusion would increase the efficacy of the combination without increasing toxicity.

The trial medication was generally well tolerated. The side effects are those predicted from the drugs used as single agents. The musculoskeletal toxicity was dose limiting in a phase I marimastat, though the plasma levels achieved at doses of 50 mg bd and above were substantially greater than those needed to inhibit MMP function *in vitro* (Wojtowicz-Praga *et al*, 1998). The dose of marimastat in this study, 10 mg twice daily, was chosen to decrease the likelihood of musculoskeletal side effects. The level of musculoskeletal toxicity was substantially below that seen in the phase I study of marimastat. Only seven patients (14%) developed grade 3 symptoms, and these responded to dose reduction or withdrawal of marimastat. The other common side effects were taste change and cough, both of which are known side effects of captopril (Havelka *et al*, 1982).

Serious toxicity was confined to haemorrhage. The three major bleeding events seen were a rectal bleed from a tumour mass, an upper gastrointestinal haemorrhage and a haemorrhagic stroke. The first two events occurred while the patients were on full dose, the latter on lower dose Fragmin. Cancer patients receiving anticoagulation therapy have a substantially increased risk of bleeding, of the order of 12% per year of treatment, compared with patients with nonmalignant disease (Prandoni *et al*, 2002). In the only published randomised study comparing prolonged LMWH treatment with conventional anticoagulation, the risk of bleeding with 3 months of LMWH treatment was lower than with warfarin therapy in patients with deep vein thrombosis (Pini *et al*, 1994). However, it is possible that the combination of Fragmin with marimastat and captopril may increase the risk of serious haemorrhage in patients with advanced cancer.

There was one PR to treatment in the trial in a patient with renal carcinoma. This patient had to stop taking marimastat due to musculoskeletal side effects. In addition, three of the total of 10 patients with renal carcinoma experienced prolonged disease stabilisation. We conclude that trial medication may have some activity in renal carcinoma. A randomised study is needed to confirm if this is the case. There was no evidence of activity in the other tumour types in the study.

The investigation of MMPIs and other biological therapies in cancer would be greatly enhanced by pharmacodynamic assays to confirm the efficacy of the drug in achieving its desired biological target effect in early phase studies (Fox *et al*, 2001). Candidate targets for MMPI activity are MMP2 and MMP9. However, in the Phase I study of marimastat, there was no inhibition of the activity plasma MMPs 2 and 9 in zymographic assays, despite achieving marimastat concentrations above that required to inhibit these enzymes *in vitro* (Wojtowicz-Praga *et al*, 1998). We therefore looked for alternative markers of MMPI activity.

We assayed serum, urine and plasma levels of proteins involved in angiogenesis. Although there were statistically significant changes in the level of Von Willibrand factor, CD105, VCAM and E Selectin during treatment, the magnitude of these effects was small, and as with the other markers there was considerable inter patient variation. The elevation of Von Willibrand factor and VCAM could reflect endothelial damage and release of these factors. The changes in CD105 and its complex with TGFb1, as well as reduction in E selectin, are compatible with an inhibitory effect on activated tumour endothelium. Likewise, while baseline levels of endostatin and activated factor VII were significantly elevated (*P*=0.03 and 0.01, respectively) at 8 weeks in patients who progressed compared with those with SD or PR, the differences were small and the degree of inter-patient variation prevents the use of endostatin and activated factor VII as predictive markers for the trial therapy.

As an alternative, we evaluated the inhibition of TNF-alpha release from PHA-stimulated lymphocytes, a process known to be inhibited by matrix metalloprotease inhibitors. Significant inhibition of TNF-alpha release from PHA-stimulated lymphocytes was seen by 24 h on the study treatment, becoming more marked after 7 days. A fall in TNF-alpha release occurred in 21 of 25 patients assayed. The degree of inhibition of TNF-alpha release varied considerably in magnitude between patients, but this assay offers a surrogate pharmacodynamic marker for this combination therapy and possibly for other MMPIs.

In conclusion, we found that the combination of marimastat, fragmin and captopril was generally well tolerated. Serious toxicity in the form of major bleeding may be attributable to fragmin. The combination may have activity in renal carcinoma, but confirmation of this would require a randomised study. We found that the measurement of levels of proteins involved in angiogenesis in the serum, plasma or urine may provide some discrimination as a pharmacodynamic marker to follow the combination therapy, but that the *ex vivo* measurement of TNF-alpha release following PHA stimulation of lymphocytes in whole blood samples may indicate activity of the combination and is worthy of further study.
